# Bidirectional modulation of Alzheimer’s disease via gut microbiota: rescue by fecal transplantation from healthy donors and aggravation by colitis-associated dysbiosis

**DOI:** 10.3389/fnins.2025.1593854

**Published:** 2025-05-23

**Authors:** Chenglong Zhou, Xin Feng, Huina Liu, Ting Cai, Yihong Li, Huadong Fan

**Affiliations:** ^1^College of Biological & Environmental Sciences, Zhejiang Wanli University, Ningbo, China; ^2^Ningbo No. 2 Hospital, Ningbo, China; ^3^Lab of Dementia and Neurorehabilitation Research, Department of Research and Development of Science and Technology, Ningbo Institute of Life and Health Industry, University of Chinese Academy of Sciences, Ningbo, China; ^4^Lab of Nanopharmacology Research for Neurodegeneration, Department of Research and Development of Science and Technology, Ningbo Institute of Life and Health Industry, University of Chinese Academy of Sciences, Ningbo, China; ^5^Innovation Center for Diagnosis and Treatment of Neurological Diseases, Ningbo Institute of Life and Health Industry, University of Chinese Academy of Sciences, Ningbo, China

**Keywords:** Alzheimer’s disease, gut-brain axis, neuroinflammation, microbiota dysbiosis, fecal transplantation

## Abstract

**Introduction:**

Emerging evidence implicates gut microbiota dysbiosis as a key modulator for the pathogenesis of Alzheimer’s disease (AD) via the gut-brain axis. To investigate the causal role of microbial communities in AD progression, we performed fecal microbiota transplantation (FMT) in APP/PS1 transgenic mice using donor microbiota from healthy wild-type mice or dextran sulfate sodium (DSS)-induced colitis mice.

**Methods:**

Cognitive function, amyloid-beta (Aβ) pathology, and pro-inflammatory cytokine levels were assessed in mice. 16S ribosomal RNA sequencing of gut microbiota and bioinformatic functional analyses were applied to identify the specific microbial communities potentially involved in AD progression.

**Results:**

FMT-WT mice (fecal microbiota transplantation from healthy wild-type mice) exhibited significant improvements in spatial memory (Morris Water Maze), exploratory behavior (Y-maze), and locomotor activity (Open Field Test), alongside reduced Aβ plaque burden and normalized expression of pro-inflammatory cytokines (IL-6, IL-1β, TNF-α) in both gut and brain tissues. Conversely, FMT-DSS mice (fecal microbiota transplantation from DSS-treated donors) displayed exacerbated cognitive deficits, heightened Aβ deposition, and elevated pro-inflammatory cytokine levels. Microbial profiling revealed stark contrasts: FMT-WT mice harbored beneficial taxa (*Bacteroides*, *Lachnospiraceae*) linked to anti-inflammatory products like short-chain fatty acid, while FMT-DSS mice showed blooms of pathogenic genera (*Erysipelatoclostridium*, *Enterobacteriaceae*) associated with neurotoxic metabolites. Functional analyses predicted enrichment of neuroprotective pathways (e.g., lysine metabolism) in FMT-WT and pro-inflammatory pathways (e.g., carbon metabolism) in FMT-DSS. Crucially, neuroinflammation occurred independently of gut barrier disruption, implicating circulating microbial metabolites as key mediators.

**Discussion:**

Our findings demonstrate that gut microbiota composition bidirectionally influences AD progression, with FMT from healthy donors attenuating neuroinflammation and pathology, while colitis-associated dysbiosis exacerbates disease hallmarks. Our study positions microbiota-targeted therapies as a promising strategy to modulate AD progression through the gut-brain axis.

## 1 Introduction

Alzheimer’s disease (AD), the most prevalent form of dementia, is characterized by widespread neuronal and glial loss driven by the accumulation of amyloid-β (Aβ) plaques and neurofibrillary tangles (NFTs) (Wang et al., 2023). While multiple hypotheses—such as the amyloid cascade, oxidative stress, cholinergic imbalance, and neuroinflammation—have been proposed to explain AD pathogenesis, accumulating evidence underscores the central role of chronic neuroinflammation in disease progression (Hampel et al., 2018; Butterfield and Halliwell, 2019; Wang H. et al., 2019). Emerging evidence reveals a critical role of dysfunctional brain white matter in the development of neurodegenerative disorders like AD (Hardy and Selkoe, 2002; Iturria-Medina et al., 2017; Nasrabady et al., 2018; Henstridge et al., 2019). Although the white matter is primarily composed of glial cells (microglia, astrocytes, and oligodendrocytes) and myelinated axons critical for CNS homeostasis, chronic neuroinflammation mediated by these cells paradoxically drives neurodegeneration and cognitive decline in Alzheimer’s disease (AD) (Wang et al., 2023). For instance, oligodendrocytes maintain axonal integrity and neuronal signaling via myelin sheath formation (Chen et al., 2021), while astrocytes support neuronal metabolism and homeostasis through nutrient provision and redox balance regulation (Chih and Roberts, 2003; Hamanaka et al., 2018; Jha and Morrison, 2018; Saito et al., 2021). Among glial cells, microglia are the resident immune cells of central nervous system (CNS) having garnered particular attention due to their pronounced production of proinflammatory cytokines (e.g., IL-1β, IL-6, TNF) in response to pathological stimuli, positioning them as primary drivers of neuroinflammation (Frost and Schafer, 2016; Colonna and Butovsky, 2017; Friedman et al., 2018; Arranz and De Strooper, 2019; Sala Frigerio et al., 2019; Leng and Edison, 2021; Mander et al., 2022).

Recent studies highlight the gut-brain axis as a key modulator of neuroinflammation in AD (Abdel-Haq et al., 2019; Needham et al., 2020; Agirman and Hsiao, 2021; Bairamian et al., 2022). Microbial metabolites, such as short-chain fatty acids and (SCFAs) and isoamylamine (IAA), influence CNS function via immune and circulatory pathways (Sampson and Mazmanian, 2015). For instance, IAA, produced by gut *Ruminococcaceae* species during aging, polarizes microglia toward a proinflammatory state, accelerating neurodegeneration in murine models (Abdel-Haq et al., 2019; Teng et al., 2022).

Therapeutic strategies for AD have shifted from solely targeting Aβ aggregation to modulating gut microbiota composition. Oral administration of GV-971, a seaweed-derived oligosaccharide, alleviated cerebral pathology and cognitive deficits in AD mice by restoring microbial balance (Wang X. et al., 2019). Similarly, fecal microbiota transplantation (FMT) from healthy donors reduced Aβ deposition and improved cognition in AD mouse models (Li et al., 2025). Despite these advances, the precise mechanisms underlying gut microbiota, intestinal inflammation, and neuroinflammation intersect in AD remain poorly defined.

In this study, we aimed to identify specific beneficial and pathogenic bacterial taxa influencing AD progression. We performed FMT in AD mice using donor feces from healthy mice (FMT-WT group) or mice with dextran sulfate sodium (DSS)-induced colitis (FMT-DSS group). Compared to controls, FMT-WT mice exhibited marked improvements in intestinal and neuroinflammation, cerebral pathology, and cognitive function, whereas FMT-DSS mice displayed exacerbated disease hallmarks. Subsequent 16S ribosomal RNA sequencing revealed distinct shifts in microbial taxa associated with AD pathology and behavior, providing novel insights into microbiota-targeted therapeutic interventions.

## 2 Materials and methods

### 2.1 Animals

Six-month-old male C57BL/6J wild-type mice and APP/PS1 double transgenic mice were housed in a controlled environment (23 ± 1°C, 55 ± 5% relative humidity) under a 12-h light/dark cycle (lights on at 8:00 AM). Food and water were provided ad libitum. All animal procedures adhered to the Guidelines for Ethical Review of Experimental Animal Welfare and were approved by the Institutional Animal Care and Use Committee (IACUC) of the Ningbo Institute of Life and Health Industry, UCAS (Protocol No. GK-2024-XM-0090; Approval Date: March 17, 2024).

### 2.2 Experimental design

After a 14-day acclimatization period, mice underwent baseline behavioral assessments [Open Field Test (OFT), Y-maze, and Morris Water Maze (MWM)]. APP/PS1 transgenic mice (*n* = 14) and C57BL/6J wild-type mice (*n* = 6) were divided into four groups: WT group: wild-type mice (*n* = 6). AD group: APP/PS1 mice treated with PBS (*n* = 4). FMT-WT group: APP/PS1 mice receiving fecal microbiota transplantation (FMT) from healthy donors (*n* = 5). FMT-DSS group: APP/PS1 mice receiving FMT from dextran sulfate sodium (DSS)-induced colitis donors (*n* = 5). Behavioral tests were repeated 1 month post-FMT. In compliance with American Veterinary Medical Association (AVMA) guidelines, mice were euthanized using CO_2_. Briefly, mice were gently placed into a euthanasia chamber without overcrowding. Next, CO_2_ was filled at an initial flow of the calculated rate of 10–30% displacement/min, and the mice were observed carefully until breathing ceases. The CO_2_ flow was maintained for 1–2 min after breathing stops. After no breathing for 5 min, the tissues (brains, colons and feces) of mice were then collected. Brain samples were processed for Aβ immunohistochemistry (IHC), while gut and brain tissues were analyzed for inflammatory cytokines. Fecal samples underwent 16S ribosomal RNA sequencing.

### 2.3 Behavioral testing

All tests were conducted between 9:00 AM and 3:00 PM by the same researcher. Mice were acclimated to the testing room for 30 min prior to each trial.

#### 2.3.1 Open Field Test

Motor activity in mice was assessed by OFT using the protocol described previously (Yoshizaki et al., 2020). Briefly, motor activity and anxiety-like behavior were assessed in a white acrylic chamber (40 × 40 × 40 cm). Mice were placed in the center, and total distance traveled (cm) and central zone entries were recorded over 10 min using a video tracking system (Amcap, StreamCam). The apparatus was sanitized with 75% ethanol between trials.

#### 2.3.2 Y-maze test

The Y-maze test (Y-maze) consists of three identical arms (A, B, and C), each measuring 50 cm in length, 25 cm in height, and 10 cm in width, arranged at 120° angles relative to one another. Prior to testing, all arms were cleaned with 70% ethanol to eliminate residual odors. Adaptation phase: mice were acclimated to the maze over 2 days, with four daily sessions. During each session, mice were placed at the distal end of arm A and allowed to freely explore all three arms for 5 min. Experimental procedure: (1) First trial set (C-arm blocked): the C-arm was blocked by placing a partition at the maze center. A food reward (animal jelly) was placed at the ends of the B- and C-arms. The mice were positioned at the distal end of arm A and permitted to explore arms A and B for 2 min. After this period, mice were temporarily returned to arm A. The partitions blocking arms A and C were removed, and mice were allowed to explore freely for an additional 2 min. Outcome criteria for this set of trial: correct exploration: entry into the previously blocked C-arm; exploration error: entry into the B-arm or failure to leave arm A within 2 min. (2) Second trial set (B-arm blocked): the B-arm was blocked using the same method, with food rewards placed at the ends of the B- and C-arms. Mice were reintroduced to arm A and allowed to explore arms A and C for 2 min. Partitions were then removed, and mice explored all arms for another 2 min. Outcome criteria for this set of trial: correct exploration: entry into the previously blocked B-arm; exploration error: entry into the C-arm or failure to leave arm A within 2 min. Termination criteria: six trial sets (three per arm configuration) were conducted daily until wild-type (WT) mice achieved an average accuracy of ≥95% (Prieur and Jadavji, 2019). After each trial, the maze was thoroughly cleaned with 75% ethanol to remove urine, feces, and residual odors, ensuring no interference between successive tests.

#### 2.3.3 Morris Water Maze

The Morris Water Maze consists of a circular tank (120 cm in diameter, 50 cm in height) filled with water maintained at 22 ± 2°C. The pool is divided into four quadrants (northwest, northeast, southeast, southwest), each marked with a distinct visual cue (square, triangle, circle, star) on the wall. A hidden platform is placed in the southwest quadrant, submerged 1 cm below the water surface. To enhance contrast for video tracking, black-furred C57BL/6J mice and APP/PS1 transgenic mice were tested in water rendered opaque using white food coloring. Training protocol: during training, mice were introduced into the pool facing the wall at one of the four quadrants. Each trial ended when the mouse located the platform and remained on it for 5 s. Escape latency (time taken to find the platform) and movement trajectory were recorded. If a mouse failed to locate the platform within 60 s, it was gently guided to the platform and allowed to remain for 10 s; in such cases, the escape latency was recorded as 60 s. After each trial, mice were dried under a heater, with a minimum 30-s interval between trials. Training spanned four consecutive days, with trials conducted across all quadrants. Probe test: on the sixth day, the platform was removed. Mice were introduced into the pool from the northeast quadrant and allowed to swim freely for 60 s (Nunez, 2008). The following parameters were measured: (1) time to first cross the former platform location, (2) number of platform crossings, and (3) time spent in the target quadrant (southwest). Increased crossings or prolonged time in the target quadrant were interpreted as stronger spatial memory retention.

### 2.4 Chronic DSS-induced colitis model in mice

C57BL/6J mice were acclimatized for 1 week and subsequently divided into two groups: a control group (*n* = 5) and a DSS (dextran sulfate sodium) group (*n* = 5). The DSS group received DSS dissolved in drinking water at an initial concentration of 1–2% (w/v) for 28 days (Huang et al., 2022). To mimic chronic colitis progression, the DSS concentration was incrementally increased by 0.5% every 5 days. Between each concentration increase, DSS was replaced with drinking water for a 2-day recovery period. The control group received drinking water throughout the 28-day experimental period. The body weight was monitored daily. Fresh fecal samples were collected to assess stool consistency (e.g., diarrhea) and occult blood using standardized scoring criteria. Disease activity was evaluated by composite scoring of three parameters: weight loss, stool viscosity, and fecal occult blood. At the endpoint, mice were euthanized, and colon tissues were excised for the subsequent analyses.

### 2.5 Fecal microbiota transplantation

Fecal suspensions for fecal microbiota transplantation (FMT) were prepared using feces collected from two groups: (1) five mice with DSS-induced colitis and (2) five healthy C57BL/6J mice. Approximately 200 mg of fresh feces were collected daily from each group, homogenized in 5 mL of sterile phosphate-buffered saline (PBS), and centrifuged at 3,000 × g for 15 min to collect the supernatant. The AD-DSS group received 200 μL of freshly prepared fecal suspension from DSS-induced enteritis mice via oral gavage daily for 4 weeks, while the AD-WT group received 200 μL of fecal suspension from healthy C57BL/6J mice under the same protocol (Zhao et al., 2021). The remaining AD and WT control groups were administered an equivalent volume of sterile drinking water via oral gavage throughout the experimental period. At the endpoint of the experiment, colon tissue, brain tissue, and fresh fecal samples were collected immediately following euthanasia and stored at −80°C for subsequent analysis.

### 2.6 Immunohistochemistry

The fixed brain tissue was sectioned at 5 μm thickness using a frozen microtome. The slices were mounted onto glass slides and stored at −20°C until use. Prior to staining, frozen sections were thawed at room temperature for 30 min and rinsed three times in phosphate-buffered saline (PBS) (5 min per wash). Endogenous peroxidase activity was blocked by incubating the sections with peroxidase-blocking solution for 10 min, followed by three additional PBS washes (5 min each). Next, antigen retrieval was performed by submerging the slides in citric acid-based antigen retrieval buffer within a microwave-safe container and heating in a microwave oven. After cooling naturally to room temperature, sections were washed three times in PBS (5 min per wash). Tissue permeabilization was achieved by incubating sections with 0.5% Triton X-100 (T8200-100 mL, Solarbio, Beijing, China) for 10 min, followed by three PBS washes (5 min each). Non-specific binding sites were blocked by encircling the tissue with a hydrophobic barrier using an immunohistochemical pen and applying 5% bovine serum albumin (BSA) to fully cover the tissue. Slides were incubated in a humidified chamber at 37°C for 2 h. Excess blocking solution was gently removed using absorbent paper. Primary antibody incubation was performed by applying β-amyloid antibody (1:400 dilution in 1% BSA; Abcam, rabbit monoclonal) onto the sections. Slides were placed in a humidified chamber and incubated overnight at 4°C. The following day, sections were washed three times in PBS (5 min per wash). A secondary antibody solution (HRP-linked Anti-Rabbit IgG, 1:400 dilution in 1% BSA; Cell Signaling Technology) was applied to the tissue, and slides were incubated horizontally in a humidified chamber at 37°C for 1 h. After three PBS washes (5 min each), freshly prepared 3,3′-diaminobenzidine (DAB) substrate solution (ZLI-9017, Shan-Golden Bridge, Beijing, China) was applied to the sections. Development was monitored under a microscope, and the reaction was stopped after 5 min by rinsing with distilled water (Fu et al., 2018). Images were captured immediately using a bright-field microscope (NE910, Nexcope, Ningbo, China).

### 2.7 RNA extraction from intestinal and brain tissues

The extraction of tissue RNA was performed by the following protocols (Zeng et al., 2024). (1) Homogenization: add 700 μL TRIzol (TransGen Biotech, Cat. No. ET111-01-V2) to a 1.5 mL microcentrifuge tube and then cut 20–30 mg of tissue into the tube and homogenize for 30 s. (2) Phase separation: add 200 μL chloroform (TransGen, Biotech, Cat. No. ET111-01-V2), vortex for 15 s, and incubate at room temperature for 2 min. Centrifuge at 12,000 × g for 15 min at 4°C. Transfer 300–400 μL of the clear upper aqueous phase to a new microcentrifuge tube. (3) RNA precipitation: add an equal volume of isopropanol (Macklin, I811925, Shanghai, China) to the aqueous phase and invert the tube 15 times to mix. Centrifuge at 12,000 × g for 10 min at 4°C. Discard the supernatant. (4) Washing: add 1 mL of 75% ethanol to the pellet. Gently resuspend the pellet by pipetting. Centrifuge at 12,000 × g for 5 min at 4°C. Discard the supernatant. Repeat with 1 mL of anhydrous ethanol. Centrifuge again under the same conditions. (5) Drying and dissolution: remove residual ethanol by blotting the tube mouth with absorbent paper. Air-dry the pellet for 20 min. Dissolve the RNA in 20 μL DEPC-treated water by pipetting 20 times gently. Finally, the RNA concentration and purity (A260/A280 ratio: 1.8–2.2) were quantified using a NanoDrop^™^ One/One^C^ spectrophotometer (Thermo Fisher Scientific, Waltham, MA, United States).

### 2.8 Reverse transcription and quantitative real-time PCR

gDNA was removed by the reaction mix of 16 μL volume containing 4 μL 4 × gDNA wiper,1 pg–1 μg RNA template mix and nuclease-free water incubating at 42°C for 2 min. Then 4 μL 5 × HiScript III qRT SuperMix (Vazyme, Cat. No. R323-01) was added to the reaction to perform reverse transcription with the following parameters: 37°C for 15 min (reverse), and 85°C for 5 s (enzyme inactivation). The cDNA product was stored at −20°C. Next, quantitative PCR was performed using qTOWER3 G System (Tianlong, Gentier 96, Xi’an, China) with the reaction volume of 10 μL containing 5 μL 2 × ChamQ Universal SYBR qPCR Master Mix (Vazyme, Q711-02, Nanjing, China), 0.2 μL forward/reverse primers (10 μM each), 2.8 μL cDNA template, and 2 μL nuclease-free water. The primer sequences are as follows: GADPH: 5’-AGGTCGGTGTGAACGGTCA-3′ (forward), 5′-TGTAGACCATGTAGTTGAGGTCA-3′ (reverse); TNF-α: 5′-CCCTCACACTCAGATCATCTTCT-3′ (forward), 5′-GCTACGACGTGGGCTACAG-3′ (reverse); IL-1β: 5′-GCAACTGTTCCTGAACTCAACT-3′ (forward), 5′-ATCTTTTGGGGTCCGTCAACT-3′ (reverse); IL-6: 5′-TAGTCCTTCCTACCCCAATTTCC-3′ (forward), 5′-TTGGTCCTTAGCCACTCCTTC-3′ (reverse). The amplification procedure was as follows: initial denaturation: 95°C for 30 s (1 cycle); cycling: denaturation: 95°C for 30 s, and annealing/extension at 65°C for 30 s (40 cycles).

### 2.9 16S ribosomal RNA sequencing of fecal samples

Total genomic DNA was extracted from fecal samples using the Mag-Bind Soil DNA Kit (Cat. No. M5635-02, Omega, MA, United States). DNA purity and concentration were assessed prior to downstream processing. The V3–V4 hypervariable regions of the 16S rRNA gene were amplified via PCR using region-specific primers with barcode sequences and a high-fidelity DNA polymerase. PCR products were resolved via 2% agarose gel electrophoresis, and target fragments (~550 bp) were excised and purified using the Quant-iT PicoGreen dsDNA Assay Kit (Thermo Fisher Scientific, P7581, MA, United States). Based on electrophoretic quantification results, purified amplicons were quantified fluorometrically using a microplate reader (BioTek Synergy HT, FLx800; Thermo Fisher Scientific). Amplicons were pooled in equimolar ratios according to sequencing requirements. Sequencing libraries were prepared using the Illumina TruSeq Nano DNA LT Library Prep Kit (Illumina, CA, United States). Library quality was verified using the Agilent Bioanalyzer 2100 system (CA, United States) and the Promega QuantiFluor (WI, United States) fluorescence quantification system. Qualified libraries were subjected to paired-end sequencing on the Illumina platform (CA, United States).

### 2.10 Data analysis

#### 2.10.1 Data processing

Raw paired-end sequencing data in FASTQ format were preprocessed using Cutadapt to identify and remove adapter sequences. Following adapter trimming, low-quality sequences were filtered out. Denoising, merging of paired-end reads, and chimera removal were performed using the DADA2 plugin in QIIME 2 (v2024.2, Northern Arizona University, United States) with default parameters. The final outputs included representative sequences for each amplicon sequence variant (ASV) and an ASV abundance table.

#### 2.10.2 Taxonomic annotation

Representative sequences for each ASV were annotated against the SILVA database (v138, 16S/18S rDNA) using the QIIME 2_classify-sklearn classifier with default settings. This step assigned taxonomic classifications to ASVs based on sequence similarity to reference entries in the database.

#### 2.10.3 Diversity analysis

α- and β-diversity metrics were computed using QIIME 2. α-diversity, reflecting within-sample microbial diversity, was assessed using indices such as observed ASVs, Chao1 and Shannon. Visualization tools included rarefaction curves and species accumulation curves to evaluate sampling depth and richness. β-diversity, comparing microbial community composition across samples, was analyzed using distance matrices (e.g., Bray–Curtis, UniFrac) and visualized via principal coordinate analysis (PCoA).

#### 2.10.4 Differential abundance analysis

LEfSe (Linear Discriminant Analysis Effect Size) was employed to identify taxa with significant differences in abundance between sample groups. This method applies linear discriminant analysis to rank taxa by effect size and performs statistical analysis (Kruskal–Wallis and Wilcoxon tests) to highlight biomarkers explaining inter-group variation. For additional validation, STAMP software was used to compare taxonomic abundances between groups via non-parametric tests (Wilcoxon for two groups; Kruskal–Wallis for multiple groups), reporting *p*-values without graphical output.

#### 2.10.5 Function prediction

PICRUSt2 (Phylogenetic Investigation of Communities by Reconstruction of Unobserved States) predicted microbial metabolic functions based on ASV abundances. Using a phylogenetic placement approach, PICRUSt2 inferred ancestral gene content from the Greengenes database (v2022.10) and projected these inferences onto the ASV phylogeny. The output provided Kyoto Encyclopedia of Genes and Genomes (KEGG) pathway abundances, enabling predictions of the microbial community’s functional potential. Additionally, detailed COG categories and pathways for functional prediction are available in the NCBI Clusters of Orthologous Genes (COGs) Database.^1^

## 3 Results

### 3.1 Fecal microbiota transplantation modulates cognitive function in AD mice

**Figure 1:**
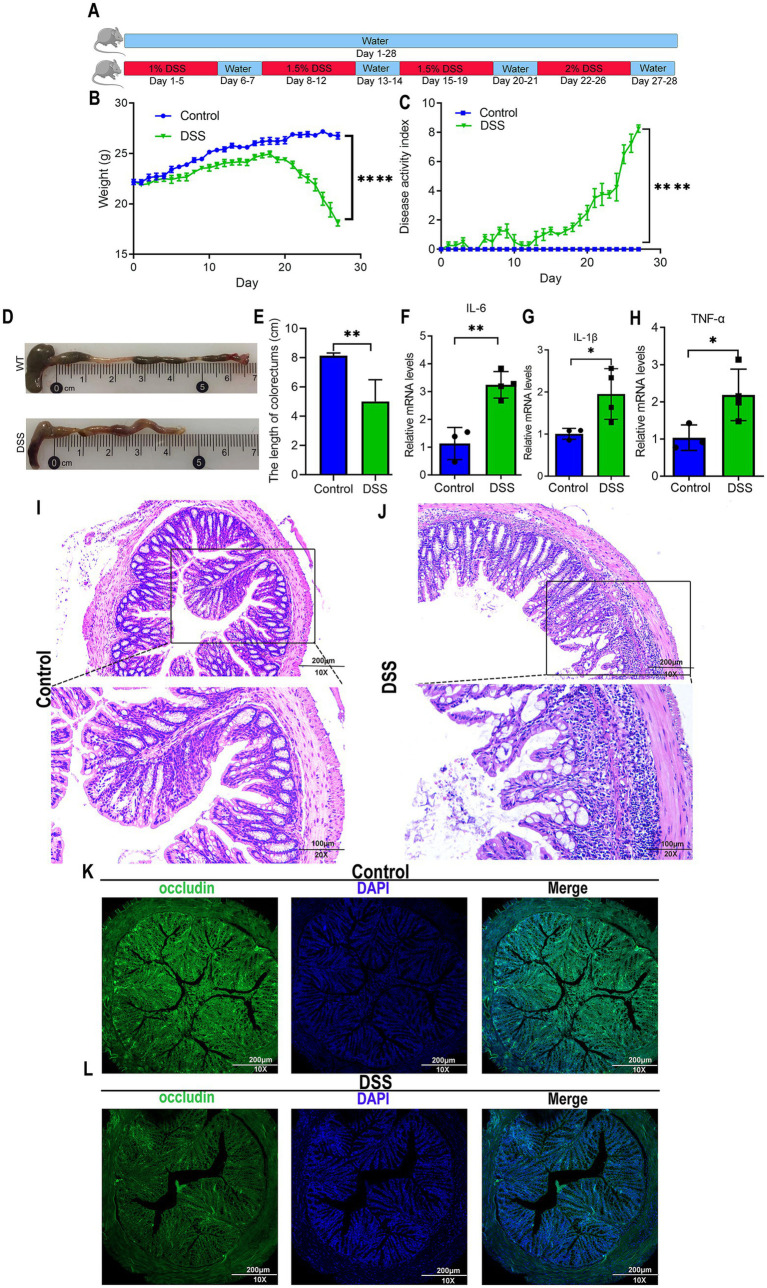
Murine model of DSS-induced colitis. **(A)** Experimental timeline for dextran sulfate sodium (DSS)-induced chronic colitis. C57BL/6J mice were divided into control and DSS-treated groups. Control mice received standard drinking water for 28 consecutive days. DSS-treated mice underwent cyclic administration: 1% DSS in drinking water for 5 days, followed by 2 days of normal water. DSS concentration increased by 0.5% in subsequent cycles (5 days DSS, 2 days water), repeated for 28 days. **(B)** Body weight fluctuations in control and DSS-treated mice. By day 20, DSS mice exhibited sharp weight loss, while control mice showed gradual weight gain. **(C)** Disease activity index (DAI) confirming successful induction of chronic colitis in DSS-treated mice. **(D,E)** Colon length measurements from control and DSS-treated mice. **(F–H)** Quantitative real-time PCR analysis of pro-inflammatory cytokine expression (IL-6, IL-1β, TNF-α) in intestinal tissues, revealing significantly elevated levels in DSS-treated mice. **(I,J)** H&E staining illustrating morphological changes in colorectal tissues. Scale bars: 200 μm for 10× magnification, and 100 μm for 20× magnification. **(K,L)** Immunofluorescence staining of occludin, a gap junction protein, in colorectal tissues, indicating a lower expression in DSS-treated mice. Scale bars: 200 μm. Data represent mean ± SEM; ^*^p < 0.05, ^**^p < 0.01, and ^****^p < 0.0001 versus control.

**Figure 2:**
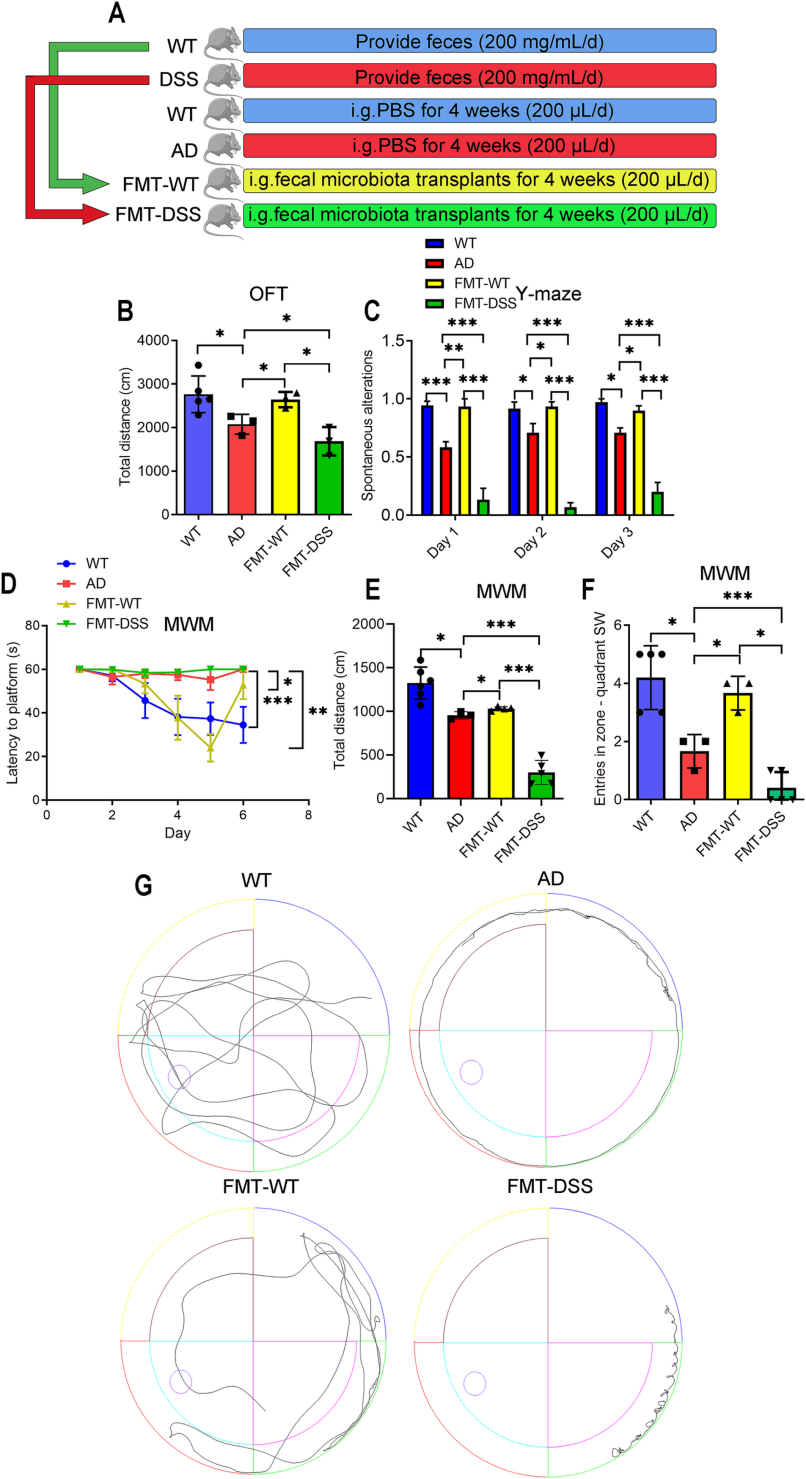
Effects of fecal microbiota transplantation (FMT) on cognitive behavior in mice. **(A)** Experimental design: healthy C57BL/6J (WT) and APP/PS1 transgenic (AD) mice were divided into four groups. AD mice received daily oral gavage (200 μL) for 4 weeks as follows: PBS (AD group), FMT from WT donors (FMT-WT group), or FMT from donors with DSS-induced colitis (FMT-DSS group). **(B)** Open Field Test (OFT) measuring total distance traveled. **(C)** Y-maze test quantifying correct arm entries. **(D–F)** Morris Water Maze (MWM) performance. **(D)** Escape latency from the NE quadrant to the SW platform. **(E)** Total swim distance. **(F)** SW quadrant entries. **(G)** Representative swim trajectories in the MWM. Data represent mean ± SEM; ^*^p < 0.05, ^**^p < 0.01, and ^***^p < 0.001.

The pathogenesis of AD is closely linked to the gut microbiota dysbiosis (Sochocka et al., 2019). Given that dextran sulfate sodium (DSS)-induced colitis serves as a well-established model for studying gut dysbiosis (Mar et al., 2014), we initiated our fecal microbiota transplantation (FMT) experiments by developing a chronic colitis model to generate a source of disrupted gut microbiota. To determine whether gut microbiota manipulation directly influences cognitive function, we performed fecal microbiota transplantation (FMT) in AD (APP/PS1 transgenic) mice using donor feces from healthy C57BL/6J mice (WT) or mice with DSS-induced chronic colitis. Successful induction of chronic colitis in DSS-treated donors was confirmed by assessing body weight, disease activity indices, colon length, and mucosal integrity (Figure 1). WT donors exhibited a balanced gut microbiota, while DSS-treated mice displayed dysbiosis, as previously reported (Nagalingam et al., 2011; Mar et al., 2014; Rangan et al., 2019; Jing et al., 2021). AD recipients of WT or DSS donor feces were designated as the FMT-WT or FMT-DSS group, respectively (Figure 2A). Four weeks post-FMT, cognitive performance was evaluated using behavioral assays: the Open Field Test (OFT, assessing locomotor activity and anxiety-like behavior; Figure 2B), Y-maze (evaluating exploratory behavior and short-term memory; Figure 2C), and Morris Water Maze (MWM, testing spatial learning and memory; Figures 2D–F). In the OFT, AD mice exhibited reduced locomotor activity (total distance moved) compared to WT mice. This deficit was significantly ameliorated in the FMT-WT group but exacerbated in the FMT-DSS group (Figure 2B). Similarly, in the Y-maze, AD mice showed elevated error rates (failure to explore novel arms), which were rescued by FMT-WT but worsened by FMT-DSS (Figure 2C). MWM results revealed impaired spatial learning and memory in AD mice, as evidenced by prolonged latency to locate the platform (Figure 2D), decreased swimming distance (Figure 2E), and reduced entries into the target zone (Figure 2F). FMT-WT mice displayed marked improvement across all above parameters, whereas FMT-DSS mice exhibited further decline. Representative swimming trajectories (Figure 2G) visually underscored these intergroup differences. Collectively, FMT from healthy donors alleviated cognitive deficits in AD mice, including motor function, exploratory behavior, and spatial memory, while FMT from colitis donors exacerbated these impairments.

### 3.2 FMT attenuates Aβ deposition in AD mouse brains

**Figure 3:**
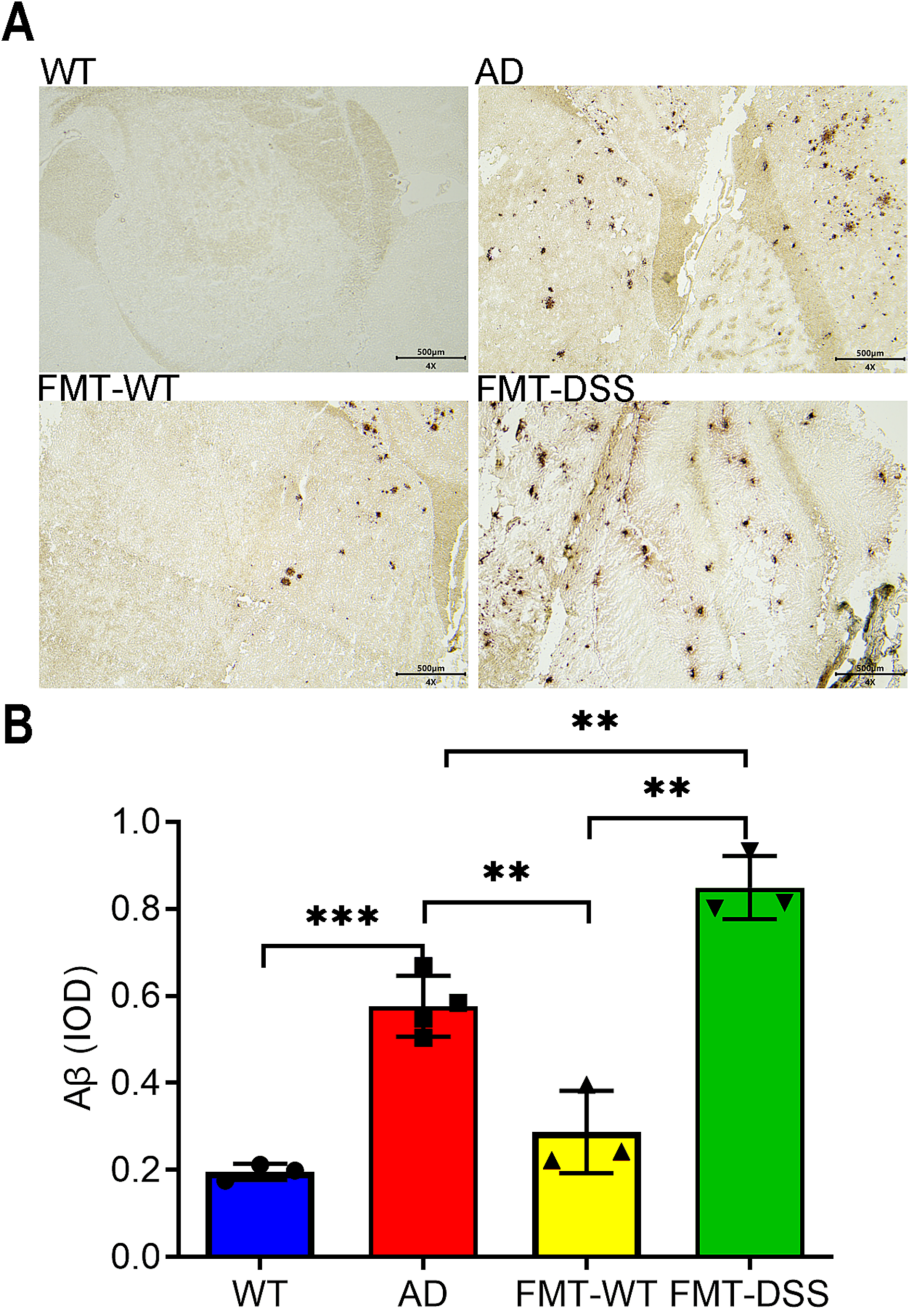
Aβ pathology in mouse brains. **(A)** Representative anti-Aβ immunohistochemical staining of brain sections. Aβ-positive deposits were observed in AD, FMT-WT, and FMT-DSS groups, but not in WT controls. Scale bar: 500 μm. **(B)** Quantification of Aβ burden using integrated optical density (IOD). Data represent mean ± SEM of three independent experiments, ^**^p < 0.01, and ^***^p < 0.001 versus control.

To assess whether gut microbiota alterations influence AD-associated neuropathology, we analyzed cerebral Aβ deposition post-FMT. Immunostaining revealed pronounced Aβ accumulation in AD mice (Figure 3A). Notably, FMT-WT mice exhibited reduced Aβ plaque intensity and distribution, correlating with their improved cognitive performance. Conversely, FMT-DSS mice displayed exacerbated Aβ pathology (Figure 3A), quantified by average optical density (Figure 3B). These findings suggest that gut microbiota modulation directly impacts Aβ burden in AD brains.

### 3.3 Gut-brain inflammatory axis mediates cognitive outcomes

**Figure 4:**
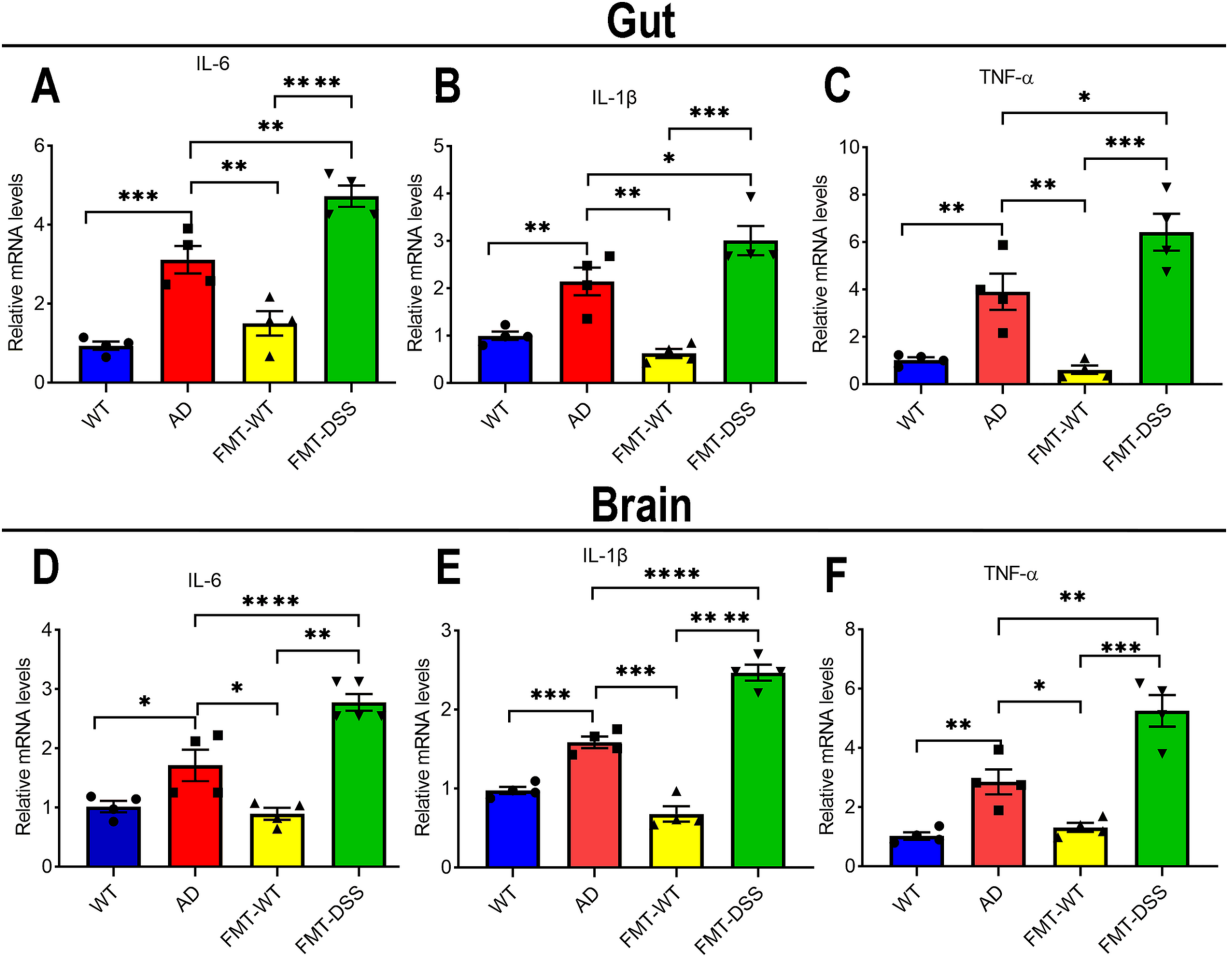
Inflammatory cytokine expression in gut and brain tissues. **(A–C)** quantitative real time PCR analysis of IL-6, IL-1β, and TNF-α mRNA levels in intestinal tissue. **(D–F)** quantitative real time PCR analysis of IL-6, IL-1β, and TNF-α mRNA levels in brain tissue. Data represent mean ± SEM of three independent experiments, ^*^p < 0.05, ^**^p < 0.01, ^***^p < 0.001, and ^****^p < 0.0001 versus control.

It’s reported that the pro-inflammatory cytokines derived from gut may finally pass the blood–brain barrier, propagating to brain parenchymal via systemic circulation (Friedman et al., 2018). To investigate whether gut-brain crosstalk is also involved in our study, we measured pro-inflammatory cytokines (IL-6, IL-1β, TNF-α) in gut and brain tissues. Gut cytokine levels were elevated in AD mice compared to WT, indicating chronic intestinal inflammation (Figures 4A–C). Interestingly, after receiving FMT from healthy donors, the chronic inflammation of gut in AD mice was significantly improved, as demonstrated by a decreased expression of these cytokines. However, FMT-DSS mice exhibited a robust increase in the expression of these cytokines compared to AD mice. Parallel trends of the pro-inflammatory cytokine levels were observed in brain tissue (Figures 4D–F), suggesting bidirectional crosstalk between gut microbiota, intestinal inflammation, and neuroinflammation.

### 3.4 FMT alters gut microbiota composition in AD mice

**Figure 5:**
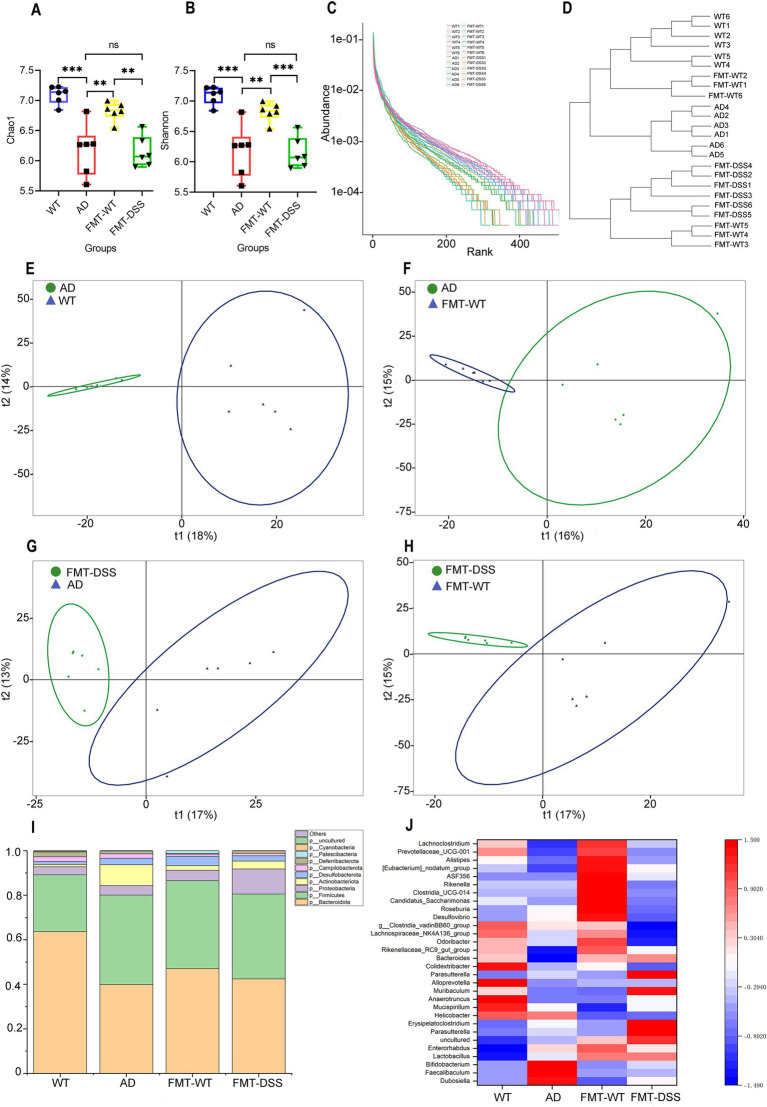
Gut microbiota diversity and composition based on 16S ribosomal RNA sequencing. **(A,B)** α-diversity indices (Chao1, Shannon) showing reduced diversity in AD and FMT-DSS groups compared to WT and FMT-WT. **(C)** Rank-abundance curves illustrating species distribution. **(D)** Bray–Curtis clustering dendrogram of microbial communities. **(E–H)** PCoA plots (weighted UniFrac) comparing β-diversity between groups. **(I)** Stacking diagram showing microbial composition. **(J)** Genus-level heatmap. Data represent mean ± SEM; ^**^p < 0.01, and ^***^p < 0.001 versus control.

Numerous studies have demonstrated a reciprocal interaction between gut inflammation and microbiota composition (Mossad et al., 2022). Given that fecal transplantation altered the inflammatory status in Alzheimer’s disease (AD) mice, we further investigated whether this intervention modulates gut microbial composition via 16S ribosomal RNA sequencing of fecal samples. A total of 24 fecal samples from four experimental groups (*n* = 6 per group) were analyzed, yielding 2,330,667 sequencing reads. After quality filtering, noise reduction, and chimera removal using DADA2 in QIIME2, 2,226,291 high-quality reads were retained. Sequences with 100% similarity were clustered into Amplicon Sequence Variants (ASVs), generating ASV counts of 3,161 (WT), 2,350 (AD), 2,944 (FMT-WT), and 2,284 (FMT-DSS). Microbial diversity within individual samples (α-diversity) was assessed using Chao1 (species richness) and Shannon (species diversity and evenness) indices. As shown in Figures 5A,B, AD mice exhibited significantly reduced Chao1 and Shannon values compared to WT mice, indicating diminished microbial abundance and diversity. Fecal microbiota transplantation (FMT) restored these indices in the FMT-WT group but not in the FMT-DSS group. Rank-abundance curves (Figure 5C) further revealed narrower horizontal spans and steeper slopes in AD and FMT-DSS groups, corroborating their lower microbial diversity and uneven species distribution compared to WT and FMT-WT groups. Hierarchical clustering (Figure 5D) demonstrated high intra-group homogeneity of the gut microbiota, with samples from the same group clustering together. In addition, β-diversity analysis via principal coordinates analysis (PCoA; weighted UniFrac distance) highlighted significant compositional differences between groups (Figures 5E–H). Notably, distinct separations were observed between WT vs. AD, AD vs. FMT-WT, AD vs. FMT-DSS, and FMT-WT vs. FMT-DSS groups. Besides diversity comparisons, we also analyzed the specific composition of gut microbial species at the phyla and genera level. At the phylum level (Figure 5I), *Bacteroidota*, *Firmicutes*, *Proteobacteria*, and *Actinobacteriota* dominated all groups. Specifically, WT and FMT-WT groups shared similar profiles, with *Bacteroidota* predominating (63.7 and 47%, respectively), followed by *Firmicutes* (25.5 and 35.5%, respectively). In contrast, AD and FMT-DSS groups showed reduced *Bacteroidota* and elevated *Actinobacteriota*. Furthermore, heatmap analysis at the genus level (Figure 5J) revealed distinct taxonomic patterns: WT mice were enriched in *Anaerotruncus*, *Mucispirillum*, *Alloprevotella*, and *Muribaculaceae*, while FMT-WT mice harbored *Desulfovibrio*, *Roseburia*, *Candidatus*_*Saccharimonas*, *Rikenella*, and *Lachnoclostridium*. AD mice exhibited dominance of *Faecalibaculum*, *Bifidobacterium*, and *Helicobacter*, whereas *Parasutterella* and *Erysipelatoclostridium* prevailed in FMT-DSS mice. These findings underscore that FMT from healthy donors could partially restore microbial diversity in AD mice, mirroring improvements in inflammation and pathology, whereas FMT from DSS donors prevents such beneficial alterations, which might be due to the compositional disparities of microbiota between FMT-WT and FMT-DSS.

### 3.5 Taxonomic differences in gut microbiota across groups

**Figure 6:**
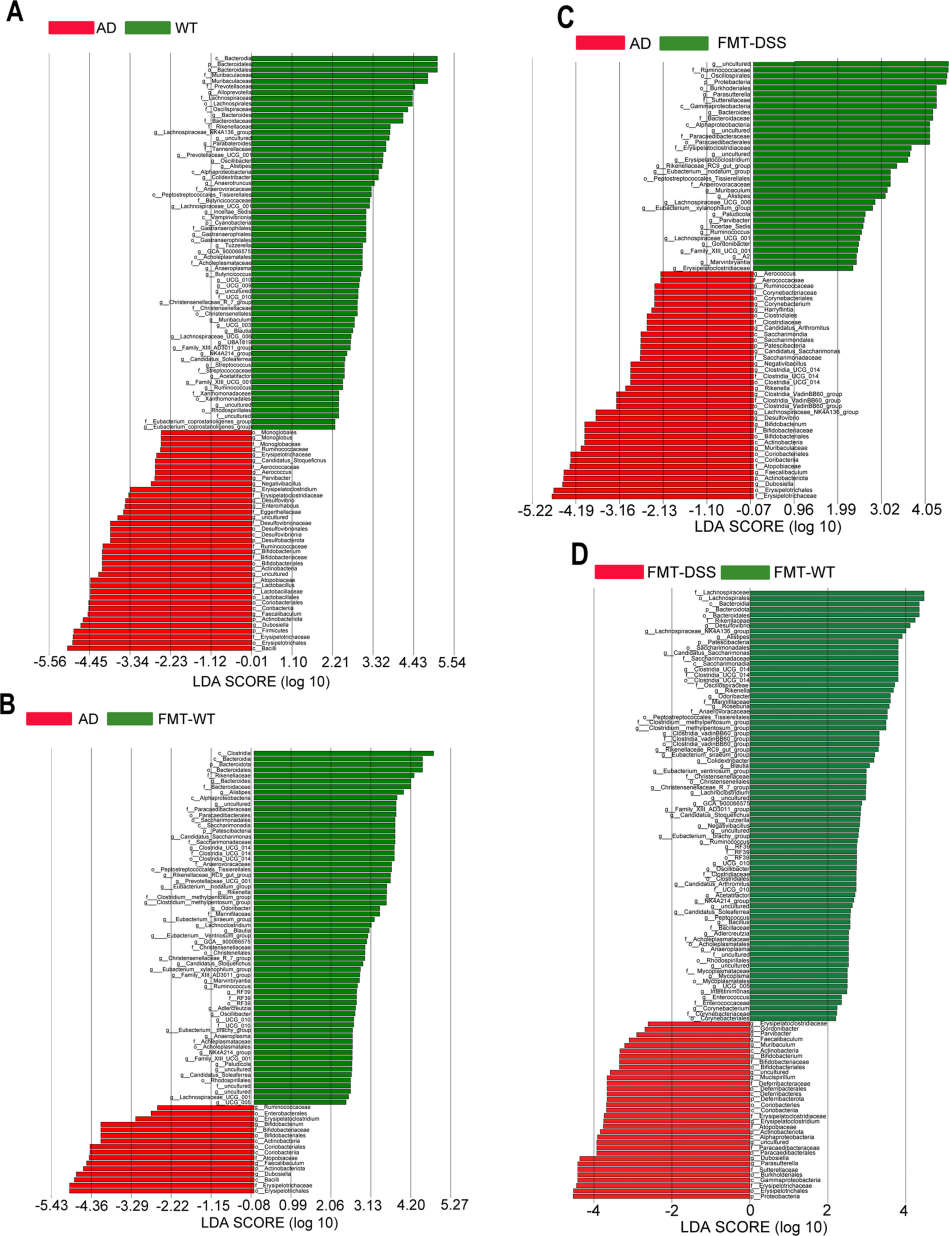
LEfSe analysis identifying differentially abundant bacterial genera. **(A–D)** Linear discriminant analysis (LDA) scores comparing WT vs. AD, AD vs. FMT-WT, AD vs. FMT-DSS, and FMT-WT vs. FMT-DSS. Column length reflects genus abundance. LEfSe, Linear Discriminant Analysis Effect Size.

To further investigate differences in microbial composition at the genus level between groups, we performed Linear Discriminant Analysis Effect Size (LEfSe) analysis. Taxa with linear discriminant analysis (LDA) scores exceeding 2.0 were considered significant for genus level discrimination. As illustrated in Figures 6A–D, comparative analyses revealed distinct taxonomic enrichments across groups: WT group: taxa such as Bacteroidetes (phylum), *Lachnospirales* (order), *Prevotellaceae* (family), and *Oscillospiraceae* (family) were significantly enriched. AD group: taxa including *Erysipelotrichales* (order), *Enterobacteriaceae* (family), *Actinobacteriota* (phylum), and *Ruminococcaceae* (family) exhibited predominant associations. FMT-WT group: taxa such as *Clostridia* (class), *Bacteroidia* (class), *Rikenellaceae* (family), and *Alistipes* (genus) were notably prevalent. FMT-DSS group: taxa including *Bacilli* (class), *Proteobacteria* (phylum), *Firmicutes* (phylum), and *Parasutterella* (genus) showed significant predominance. These results highlight group-specific microbial signatures, underscoring taxonomic shifts linked to experimental conditions.

### 3.6 Functional analysis of gut microbial composition

**Figure 7:**
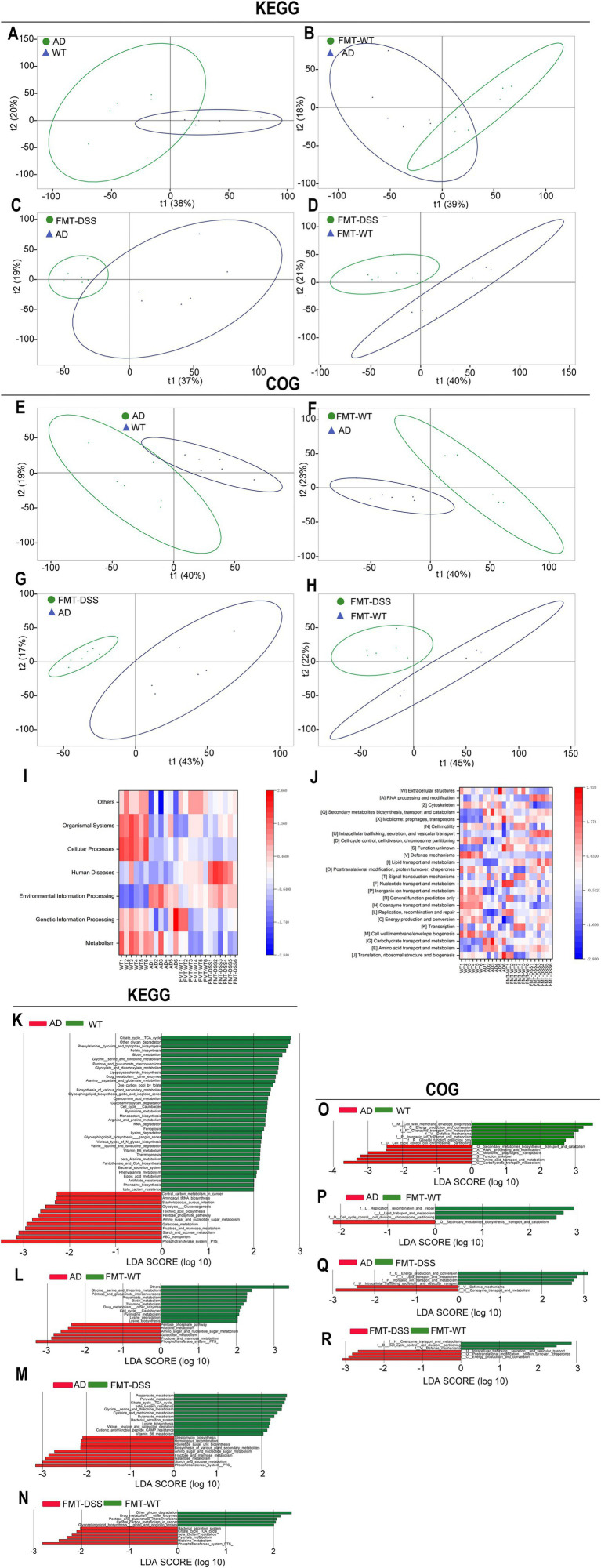
Functional analyses of gut microbiota. **(A–D)** Principal component analysis (PCA) of KEGG pathways across groups. **(E–H)** PCA of COG categories. **(I,J)** Heatmaps of KEGG and COG functional predictions. The capital letters in square brackets correspond to COG categories; for example, [W] denotes the “Extracellular structures” category. **(K–N)** LEfSe analysis of KEGG pathways. **(O–R)** LEfSe analysis of COG categories. KEGG, Kyoto Encyclopedia of Genes and Genomes; COG, Clusters of Orthologous Genes.

Based on the observed compositional differences in gut microbiota across mouse groups, we hypothesized that these variations might lead to divergent metabolic functions. As 16S rRNA sequencing identifies taxonomic profiles but not direct gene functions, we employed PICRUSt, a bioinformatics tool, to infer metabolic potential by mapping sequencing data to the KEGG (Kyoto Encyclopedia of Genes and Genomes) and COG (Clusters of Orthologous Genes) databases. KEGG analysis can provide a systems-level view on metabolic pathways (e.g., amino acid synthesis), while COG analysis focuses on protein-specific functions (e.g., membrane transporters) predicting functional characters at the protein level. As shown in Figures 7A–D, β-diversity analysis of KEGG pathways revealed significant distinctions between the following groups: WT vs. AD, FMT-WT vs. AD, FMT-DSS vs. AD, and FMT-DSS vs. FMT-WT. Similarly, COG-based β-diversity analysis showed marked differences between the same group pairs (Figures 7E–H). Furthermore, heatmap visualization of KEGG and COG profiles highlighted group-specific functional enrichments, as depicted in Figures 7I,J, the WT group exhibited enrichment in pathways related to body defense mechanisms and core metabolic processes. In contrast, the AD group showed significant enrichment in functions linked to environmental signal transduction, along with biosynthesis, transport, and catabolism of secondary metabolites. The FMT-WT group displayed predominant pathways associated with genetic information processing and transcriptional regulation, while the FMT-DSS group showed notable enrichment in pathways pertaining to human diseases, lipid transport, and metabolic dysregulation. To further delineate functional disparities, LEfSe-based linear discriminant analysis (LDA) was performed on KEGG and COG profiles (Figures 7K–R). The WT group demonstrated enrichment in defense mechanisms, coenzyme metabolism, cell cycle regulation, and amino acid biosynthesis. The AD group exhibited heightened activity in carbon metabolism, thiocyanate biosynthesis, pentose phosphate pathway, and *Staphylococcus aureus* infection-related pathways. The FMT-WT group was markedly overrepresented with metabolic pathways tied to lysine and pyrimidine metabolism, whereas the FMT-DSS group showed activation of oncogenic carbon metabolism, peptidoglycan biosynthesis, glutathione metabolism, and pathways linked to *Staphylococcus aureus* infection.

## 4 Discussion

Alzheimer’s disease (AD) is a progressive neurodegenerative disorder characterized by neuroinflammation, amyloid-β (Aβ) deposition, and cognitive impairment. Growing evidence underscores the gut-brain axis as a pivotal regulator of AD pathogenesis, with dysbiosis of gut microbiota linked to systemic and central nervous system (CNS) inflammation. In this study, fecal microbiota transplantation (FMT) from healthy donors or those with colitis significantly modulated cognitive behavior (Figure 2), Aβ pathology, and inflammatory cytokine profiles in APP/PS1 transgenic mice (Figures 3, 4). These results emphasize the therapeutic promise of gut microbiota modulation in alleviating AD-related neurodegeneration. Mechanistically, this effect may arise through sequential pathways: shift in microbiota composition drive changes in microbial metabolites production, which subsequently influence neuroinflammatory signaling. This process can be explained in detail as follows: (1) Potential mechanisms by which FMT from healthy donors alleviates AD: fecal microbiota transplantation from healthy donors to AD mice (FMT-WT) showed enrichment of *Bacteroides* and *Lachnospiraceae* (Figures 5, 6), genera linked to with anti-inflammatory short-chain fatty acids (SCFAs) such as butyrate (Erny et al., 2015; Dalile et al., 2019; Mossad et al., 2022). Butyrate can suppress microglial activation by inhibiting histone deacetylases (HDACs), thereby reducing pro-inflammatory cytokine production (Stilling et al., 2016; Matt et al., 2018; Chen et al., 2019). Additionally, *Bacteroides thetaiotaomicron*, also enriched in FMT-WT mice, metabolize dietary glucosinolates into sulforaphane (SFN), a potent anti-inflammatory compound (Liou et al., 2020). SFN can cross the blood–brain barrier (BBB) to activate Nrf2 pathways, mitigating oxidative stress in microglia (Tarozzi et al., 2013; Zhang et al., 2015). SFN, a key metabolite of *Bacteroides thetaiotaomicron* with antioxidant, anti-inflammatory and anti-apoptotic properties, has demonstrated therapeutic promise in neurodegenerative diseases, including AD, Parkinson’s disease (PD), and multiple sclerosis (MS) (Schepici et al., 2020). Furthermore, *Bacteroides fragilis* produces polysaccharide A (PSA)-containing outer membrane vesicles (OMVs), which induce immunomodulatory effects and attenuate experimental colitis (Shen et al., 2012), suggesting a potential role in suppressing neuroinflammation. While direct evidence linking these species to AD pathogenesis remains limited, our findings indicate that FMT from healthy donors introduces a consortium of bacteria producing diverse neuroprotective metabolites, surpassing the narrow scope of single-compound therapies like GV-971 (Wang X. et al., 2019). Collectively, the shift from pathogenic to beneficial bacteria in FMT-WT mice likely reduces microglial hyperactivation, highlighting the role of microbial metabolites in modulating neuroinflammation. (2) Potential mechanisms by which FMT from colitis donors exacerbates AD: microglia, the resident immune cells of the CNS, play dual roles in AD. While they initially clear Aβ plaques via phagocytosis, chronic activation drives neuroinflammation through the release of pro-inflammatory cytokines (IL-1β, IL-6, TNF-α) and reactive oxygen species (ROS), exacerbating neuronal damage (Heneka et al., 2015; Hickman et al., 2018). Our results align with this paradigm: AD mice exhibited elevated cytokine levels in both gut and brain tissues, correlating with cognitive deficits and Aβ deposition. Notably, FMT from healthy mice (FMT-WT) reduced neuroinflammation and Aβ burden, whereas FMT from colitis mice (FMT-DSS) worsened these parameters (Figure 3), suggesting gut microbiota composition directly influences microglia-mediated neuroinflammation and neurodegeneration. A potential trigger of this interaction is isoamylamine (IAA), a metabolite produced by gut *Ruminococcaceae* species (Teng et al., 2022). Teng et al. (2022) demonstrated that IAA promotes microglial cell death via mitochondrial dysfunction, accelerating age-related cognitive decline. In line with this, our 16S rRNA sequencing revealed a robust increase of *Ruminococcaceae* abundance in AD and FMT-DSS groups (Figures 5, 6), potentially explaining heightened neuroinflammation. Similarly, *Erysipelatoclostridium*, enriched in FMT-DSS mice, generates trimethylamine (TMA), which is converted to trimethylamine-N-oxide (TMAO) in the liver, a pro-atherogenic molecule linked to BBB disruption and microglial activation in AD (Tang et al., 2013; Vogt et al., 2017; Mossad et al., 2022). Additionally, *Parasutterella*, enriched in FMT-DSS mice (Figures 5, 6), produce succinate, a driver of Th17 cell differentiation and IL-17-mediated neuroinflammation (Rubic et al., 2008; Schneider et al., 2018; Agirman and Hsiao, 2021; Zhang et al., 2024).

One unexpected finding in our study was the absence of gut barrier damage in AD and FMT-DSS mice (data not shown), despite elevated pro-inflammatory cytokines. This contrasts with prior studies implicating “leaky gut” in neuroinflammation, where compromised intestinal permeability allows bacterial lipopolysaccharides (LPS) and metabolites to enter systemic circulation (Maes et al., 2008; Fasano, 2012; Braniste et al., 2014). Our results suggest that neuroinflammation in AD may instead propagate through alternative pathways: (1) Circulatory signaling: pro-inflammatory cytokines (e.g., IL-6) released by gut-resident immune cells can traverse the blood-brain barrier (BBB) through saturable transport systems (Banks, 2005). These cytokines prime microglia to adopt a pro-inflammatory phenotype, amplifying Aβ-induced neuronal damage. (2) Immune cell trafficking: gut-derived dendritic cells and T cells may migrate to the brain, carrying microbial antigens that directly activate microglia (D’Mello and Swain, 2017; Agirman and Hsiao, 2021). (3) Systemic inflammation: while vagus nerve signaling is a recognized gut-brain communication pathway (Goehler et al., 2000; Agirman and Hsiao, 2021), our data imply it plays a minor role here. Instead, systemic inflammation appears to be the primary driver. The preserved gut barrier in our model suggests that microbial metabolites, rather than whole bacteria or LPS, are critical mediators of neuroinflammation. For instance, aging-associated metabolites such as *N*(6)-carboxymethyllysine (CML), a product of gut microbial glycolysis, accumulates in AD brains. CML promotes microglial oxidative stress and mitochondrial dysfunction, as demonstrated by Mossad et al. (2022). Although we did not directly measure CML levels, functional KEGG pathway analysis (Figure 7) revealed enrichment of “carbon metabolism” and “pentose phosphate pathway” in FMT-DSS mice, processes linked to advanced glycation end-products (AGEs) like CML. Notably, chronic gut microbiota dysbiosis in aging is associated with gradual gut barrier deterioration and sustained metabolite leakage (Takeuchi et al., 2000; Srikanth et al., 2011). However, our chronic FMT-DSS model likely introduced a surge of pre-formed metabolites without immediate barrier damage. This distinction underscores the temporal dynamics of gut-brain interactions: early-stage dysbiosis may depend on soluble mediators, whereas chronic stages involve structural gut damage. Thus, even with an intact gut barrier in our model, circulating harmful metabolites can still exacerbate neurodegeneration.

The 16S rRNA sequencing data revealed stark contrasts in microbial composition between groups. FMT-WT mice were enriched with *Bacteroides thetaiotaomicron* and *Bacteroides fragilis*, both exhibiting therapeutic potential: (1) *Bacteroides thetaiotaomicron* metabolizes dietary glucosinolates into SFN, which enhances glutathione synthesis to mitigate oxidative stress (Liou et al., 2020). Our ongoing research demonstrates that broccoli-derived exosomes (BDELNs) rich in SFN reduce Aβ plaques in AD mice; however, their efficacy depends on *Bacteroides thetaiotaomicron* for SFN bioactivation (Liou et al., 2020; Li et al., 2024). (2) *Bacteroides fragilis* secretes PSA-containing OMVs, which suppress colitis by inducing IL-10-producing regulatory T cells (Tregs) (Shen et al., 2012). In the brain, PSA may similarly dampen microglial activation, suggesting a dual protective mechanism targeting both gut and brain inflammation. In contrast, FMT-DSS mice exhibited marked increases in *Erysipelatoclostridium* and *Enterobacteria*, genera linked to LPS and TMAO production, metabolites related with neuroinflammation and neurodegeneration.

The DSS-induced colitis mouse model used here is well-established, with prior studies demonstrating that DSS treatment reproducibly induces dysbiosis characterized by blooms of pro-inflammatory taxa (e.g., *Enterobacteriaceae*, *Erysipelatoclostridium*) and depletion of beneficial SCFA-producing genera (e.g., *Bacteroides*, *Lachnospiraceae*) (Nagalingam et al., 2011; Mar et al., 2014). Hence, while we did not sequence the microbiota of colitis donors, we rigorously compared the FMT-WT (healthy donor) and FMT-DSS (colitis donor) recipient groups. FMT-DSS group showed microbial shifts consistent with colitis donor profiles, including increased *Ruminococcaceae* (linked to neurotoxic metabolites) and *Erysipelatoclostridium* (producer of TMAO precursor) (Mar et al., 2014; Mossad et al., 2022). FMT-DSS group also mirrored the dysbiosis features of colitis donors, showing reduced α-diversity, increased pathogenic genera (e.g., *Erysipelatoclostridium*), and pro-inflammatory pathway enrichment (Figures 5–7).

While we characterized the recipient (FMT-DSS) microbiota and observed compositional shifts consistent with prior DSS-induced dysbiosis, the lack of direct donor sequencing prevents definitive confirmation of the transferred microbial profile. This limitation underscores the importance of future studies incorporating donor microbiota sequencing to directly link specific donor taxa to recipient phenotypes. Such data would enable rigorous tracking of microbial transfer efficiency and identification of causal taxa driving AD exacerbation. Additionally, metabolomic profiling of donor fecal samples could clarify whether pre-formed neurotoxic metabolites (e.g., TMAO) were directly transplanted, further elucidating mechanisms. Furthermore, future experiments should validate donor-recipient microbial consistency and explore whether standardizing donor microbiota (e.g., via gnotobiotic models) enhances translational relevance. By addressing these gaps, subsequent studies can refine microbiota-targeted interventions for AD, ensuring robust causal inferences and therapeutic reproducibility.

In summary, our study demonstrates that FMT can bidirectionally modulates AD progression by reshaping gut microbiota and their metabolic output. The key findings include: (1) Therapeutic potential of beneficial microbiota: the FMT-WT group (transplanted with healthy microbiota) showed marked improvement, underscoring the value of beneficial bacteria such as *Bacteroides* and *Lachnospiraceae* in mitigating AD pathology. (2) Risks of pathogenic dysbiosis: conversely, FMT-DSS (transplanted with colitis-associated microbiota) exacerbated AD symptoms, highlighting the dangers of dysbiosis dominated by pro-inflammatory taxa. (3) Mechanistic insight: we challenge the prevailing “leaky gut” hypothesis by showing that neuroinflammation in AD can occur despite an intact gut barrier, emphasizing circulating microbial metabolites (e.g., SCFAs, TMAO) as primary mediators. These results suggest that AD therapeutic strategies should prioritize microbial metabolite profiling alongside barrier integrity assessments. For example, antibiotics targeting pathogens (e.g., *Enterobacteria*) or prebiotics enhancing SCFA production could benefit AD patients without pre-existing gut damage. Future studies should focus on metabolite quantification (e.g., SFN, TMAO) and combinatorial therapies targeting specific bacterial taxa.

## Data Availability

The data presented in the study are deposited in the Sequence Read Archive (SRA) of NCBI repository, accession number PRJNA1263742 (https://www.ncbi.nlm.nih.gov/bioproject/PRJNA1263742).

## Footnotes

^1^ https://www.ncbi.nlm.nih.gov/research/cog/#
